# Electrocardiogrammes d´un patient COVID-19 traité par l´association hydroxychloroquine et azithromycine

**DOI:** 10.11604/pamj.supp.2020.35.2.23878

**Published:** 2020-07-14

**Authors:** Mohamed Elghali Benouna, Amine Ech-chenbouli

**Affiliations:** 1 Département de Cardiologie, Rue des hôpitaux, CHU IbN Rochd, Casablanca, Maroc

**Keywords:** COVID-19, hydroxychlroquine, azithromycine

## Image en medicine

Un patient de 73 ans a été admis dans notre hôpital pour prise en charge d´une forme modérée d´infection au COVID-19 découverte au décours d´un syndrome coronaire aigu revascularisé par un stent de la Circonflexe à 8h de la douleur thoracique. Il avait comme antécédent un diabète de type 2 sous Metformine et une hypertension artérielle sous Périndopril. Après un ECG initial montrant un Qt (corrigé selon la formule de Bazett) à 420ms, le patient a été mis sous hydroxychloroquine 200mg x3/j et azithromycine 500mg/j conformément au Protocol marocain de traitement des patient COVID-19 en plus de son traitement post infarctus: clopidogrel 75mg 1cp/j; aspirine 75mg 1cp/j; atorvastatine 80mg 1cp/j, bisoprolol 2.5 mg/j. Au 5^e^ jour de son hospitalisation il a présenté sur l’ECG quotidien de surveillance un prolongement du QT (corrigé selon la formule de Bazett) devenu à 547 ms avec une extrasystole ventriculaire (ECG A) justifiant l’arrêt de l’association hydroxychloroquine/ azithromycine. Trois heures après un autre ECG a objectivé le passage en tachyatriale focale à conduction variable avec un QTc inchangé (ECG B). Il n´y avait pas de troubles hydro électrolytique associés avec une kaliémie à 4.1 mmol/l et une magnésémie à 22mmol/l. L´évolution a été favorable avec retour du QT aux normes et passage du patient en fibrillation auriculaire paroxystique nécessitant une anticoagulation orale et une sortie du patient au bout de 21 jours d´hospitalisation.

**Figure 1:**
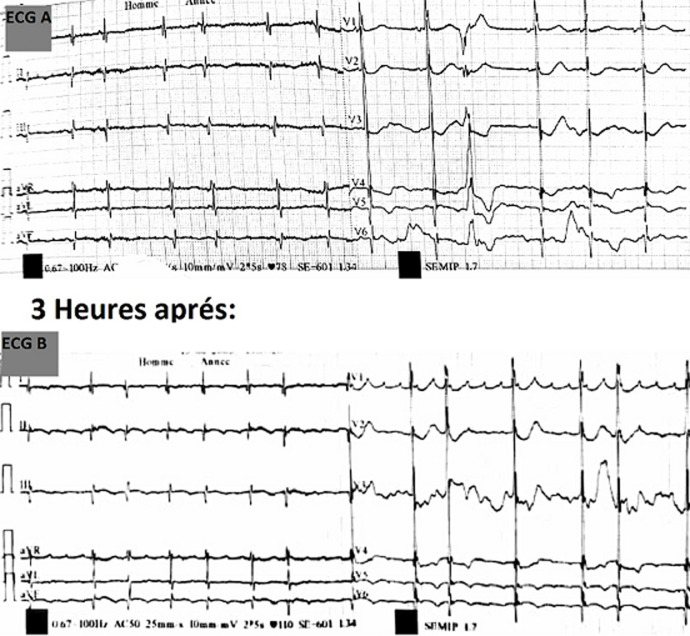
electrocardiogramme 12 dérivations objectivant un Qt allongé et extrasystole ventriculaire (A) tachycardie atriale focale (B)

